# Marginal mandibulectomy in the surgical treatment of tonsil and retromolar trigone tumours

**DOI:** 10.1016/S1808-8694(15)31064-8

**Published:** 2015-10-22

**Authors:** Maria Beatriz Nogueira Pascoal, José Francisco Chagas, Nivaldo Alonso, José Luiz Aquino, Marcus Castro Ferreira, Maria Isabel Nogueira Pascoal, Luiz A Magna

**Affiliations:** 1 Doctoral student in medicine at the Plastic Surgery Department at USP - Sao Paulo, Assistant Surgeon of the Head & Neck Surgery Unit at PUCCAMP.; 2 Doctor in medicine trained at UNIFESP, Chief of the Head & Neck Surgery Unit at PUCCAMP.; 3 Lecturer on medicine at USP - Sao Paulo, Associate Professor of the Plastic Surgery Discipline at USP-SP.; 4 Doctor in medicine trained at UNICAMP, Assistant Surgeon in the Head & Neck Surgery Unit at PUCCAMP.; 5 Lecturer at USP - Sao Paulo, Full Professor of the Plastic Surgery Discipline at USP - Sao Paulo.; 6 Master in Head & Neck Surgery trained at Heliopolis - Sao Paulo, Responsible for Bucomaxillofacial Prosthetic Rehabilitation in the Head & Neck Surgery Unit at PUCCAMP.; 7 Doctor in medicine trained at UNICAMP, Adjunct Professor of the Genetics Department at UNICAMP.; 8 Head & Neck Surgery Unit at the Campinas Pontifical Catholic University, Plastic Surgery Department of the USP Medical School - SP.

**Keywords:** mandibulectomy, mandibulotomy, oral tumours, oropharyngeal tumours

## Summary

**R**esection of the ascending ramus of the mandible can result in considerable functional and esthetic damage. **Aim:** To compare the survival rate and local and regional recurrence in marginal and segmental mandibulectomy for advanced tonsil and retromolar trigone tumours with no mandibular invasion. PLACE AND PERIOD: Reference Head & Neck Service, between October 1994 and December 2001. **Material and method:** 20 stage IV patients undergoing marginal mandibulectomy and 22 undergoing segmental mandibulectomy were compared. **Case study:** a contemporary cross-sectional cohort study. **Results:** Of 20 patients undergoing marginal mandibulectomy, 35% died of the disease, 15% due to local recurrence, 15% due to regional recurrence and 5% due to local and regional recurrence. Of 22 patients undergoing segmentary mandibulectomy, 36,4% died of the disease, 31,8% due to local recurrence and 13,6% due to distant recurrence. The Kaplan-Meier analysis showed a 55% survival rate for the marginal mandibulectomy group, and a 45% survival rate for the segmental group (p= 0.8329). **Conclusions:** Analysis of the two groups showed that conservation of the ascending ramus of the mandible, even in advanced lesions with no mandibular involvement, does not increase the recurrence rate.

## INTRODUCTION

In past decades resection of the ascending ramus of the mandible has been considered as obligatory in the treatment of retromolar space and tonsillar region tumors. Although necessary at times, this causes functional and esthetic loss, frequently with irreparable damage to the patient's quality of life. Marginal resection of the mandibular bone was developed as a feasible therapeutic alternative, where a segment of the mandibular ramus is maintained in the treatment of lesions with no bone involvement. In this case the recurrence rate is not increased, and the principles of oncological radicality are not compromised. This paper aims to analyze the survival rate and local/regional recurrence in resection of advanced retromolar space and tonsillar region tumors where the treatment involved marginal resection of the mandibular bone.

## SERIES AND METHOD

A retrospective analysis was made of 42 patients diagnosed with epidermoid carcinoma between October 1994 and December 2001. There were 39 men and 3 women aged between 36 and 72 years (mean: 50.5 years; median: 50.7 years). Inclusion criteria were patients with advanced lesions involving the tonsillar region or the retromolar space, and a panoramic radiographic study or a computed tomography demonstrating no mandibular bone involvement. Advanced disease was defined based exclusively on the size of the lesion regardless of cervical lymphnode involvement. Patients that had undergone radiotherapy or surgical treatment were excluded from the study.

There were 31 patients (73.8%) with tonsillar region lesions and 11 patients (26.2%) with retromolar space lesions. According to the UICC-2002 classification table for malignant tumors, 28 patients (66.7%) were staged IVa (patients with lesions that invaded deep/extrinsic muscles of the tongue, the pterygoid muscle, or the hard palate) and 14 patients (33.3%) were staged IVb (patients with invasion of the lateral pterygoid muscle, or pterygoid laminae, or the lateral wall of the nasopharynx), as shown on Table 1. The study was approved by the Research Ethics Committee (FR 510/05; CAAE 1017.0.1.47.000-05).

**Table 1:** Distribution of patients according to clinical and surgical staging.

	N0	N1	N2a	N2b	N2c	N3	TOTAL
T4a	12	11	0	2	2	1	28
T4b	3	5	1	2	1	2	(66,7%)
TOTAL	15	15	1	4	3	3	14
(%)	35,7	35,7	2,3	9,5	7,5	7,1	(33,3%)

**Figure 1:**
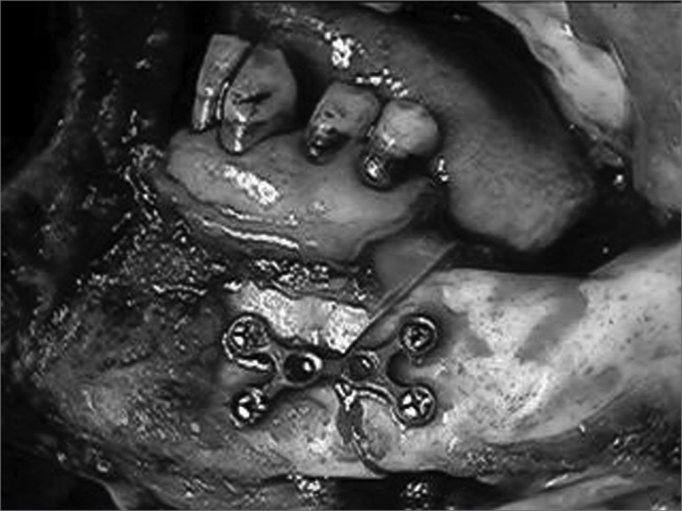
Mandibulotomy associated with marginal mandibulectomy in tonsillar region lesions.

The series was divided into two groups. The first group contained 20 patients that were treated by marginal resection of the mandibular bone. Fourteen of these patients were stage T4a and 6 patients were stage T4b. The second group (control group) consisted of 22 patients treated by segmental resection of the mandibular bone. Fifteen of these patients were T4a and 7 were T4b. All of the patients underwent neck dissection; reconstruction was done by primary closure in 38 patients and by a pectoralis major myocutaneous flap in 4 patients. All of the patients underwent postoperative radiotherapy since these were advanced tumors and/or presented neck metastases. No patients were treated with preoperative or postoperative chemotherapy. In patients treated by marginal mandibulectomy the procedure started with a paramedian access mandibulotomy, angled below the emergence of the mentonian nerve. Marginal mandibulectomy was done from the body of the mandible to the coronoid apophysis above the mandibular canal (Figure 1).

**Figure 2:**
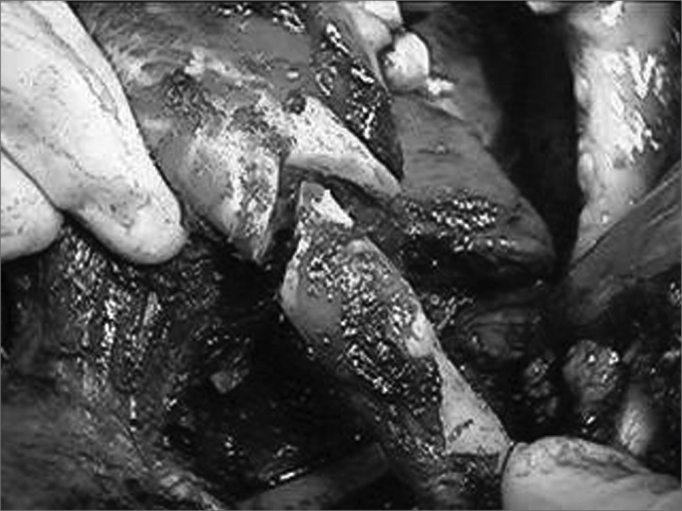
Ostheosynthesis using titanium miniplates.

**Figure 3:**
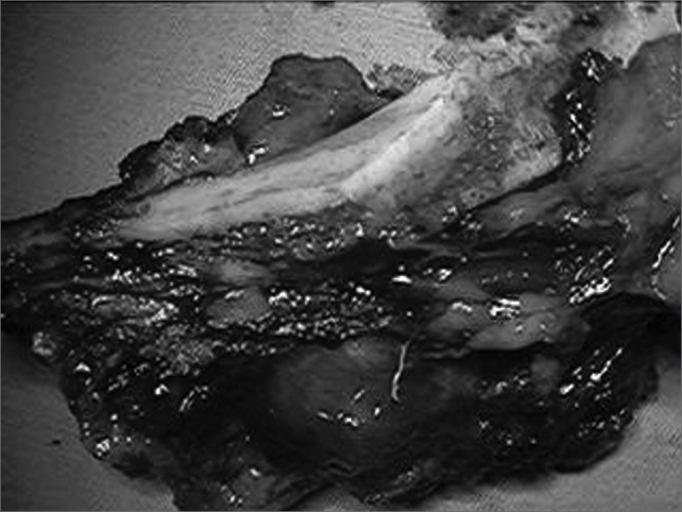
Highlight of mandibular margins and the surgical specimen.

Ostheosynthesis with 2.4 mm thickness titanium miniplates and bicortical screw fixation was used to reconstruct the mandibular arch (Figures 2 and 3). A segmental mandibulectomy was done by sagittally sectioning the ascending ramus and the mandibular body, limited by the mentonian foramen.

A single team of pathologists analyzed the surgical specimens. Formic acid at 0.07% was used to decalcify small resected bone segments, and nitric acid at 1% was used to decalcify larger resected bone fragments. Specimens were hematoxilin-eosin stained.

Actuarial survival analysis using the Kaplan-Meier method was used for both groups, which were also compared statistically using the non-parametric log rank test.

## RESULTS

Follow-up of 20 patients treated with marginal mandibulectomy was from 9 to 60 months. Thirteen patients (65%) were followed-up during 30 months, and 8 patients (40%) were followed-up during 60 months. There were two immediate postoperative deaths, one due to lung thromboembolism and one due to a second lesion on the posterior wall of the hypopharynx. This second patient survived 31 months, underwent total pharyngolaryngectomy and reconstruction using a greater curvature gastric tube, and eventually died of sepsis. Seven patients (35%) died with the disease, 3 due to local recurrence, 3 due to regional recurrence, and 1 due to local/regional recurrence. Minimum survival time was 9 months. Eleven patients (55%) are disease-free to this day. Free margins were found around all of the surgical specimens, but two patients had close surgical margins (less than 1 cm), one of them a patient that died due to local recurrence. Lymphnode metastasis was found in the surgical specimens of 15 patients, four of them with extracapsular rupture. All of the patients that had regional recurrence had extracapsular rupture. The average number of dissected lymphnodes per surgical specimen was 40, and the average number of dissected lymphnodes with invasion was 5 per patient. Analysis of the mandibular bone above the mandibular canal showed no signs of tumor involvement. Local and regional control at 30 months was 63.1%, and at 60 months was 57.8%, both excluding the patient that died immediately after surgery and the patient that died of a second tumor after surviving 31 months.

Follow-up was 14 to 60 months for the 22 patients treated with segmental resection of the mandibular bone, of which 12 (54.5%) had a follow-up period over 30 and 60 months. Eleven patients (50%) are currently alive and disease-free; 10 patients (45.4%) died due to the disease, of which seven due to local recurrence and three due to distant recurrence. One disease-free patient died due to other causes, and one patient died immediately after surgery. The local and regional control rate was 63.6%, excluding patients with local recurrence. Free margins were obtained in 20 patients, and close surgical margins were found in three patients, of which one died due to local recurrence. Lymphnode metastases were found in 12 patients of the series, an average 7 lymphnodes per patient; extracapsular rupture was seen in 7 patients. The three patients with disease extension at a distance had extracapsular rupture and extension of the tumor. The average number of dissected lymphnodes for each cervical dissection was 43.

The actuarial survival curve based on the Kaplan-Meier method showed that the group treated with marginal mandibulectomy had a 55% survival rate in a 25 to 41 month interval, a 4-month standard error, and a 95% confidence level. The group treated with segmental resection had a 45% survival rate, in a 17 to 38 month interval, a 4-month standard error, and a 95% confidence level. Both curves were compared using the Log Rank non-parametric test at p=0.8329, and the difference was considered non-significant.

## DISCUSSION

The surgical treatment of tonsillar region and retromolar space tumors started in 1940, and was based on compound resection of lesions including the complete mandibular bone, and treatment of cervical lymphnodes. At the time segmental resection of the ascending ramus of the mandible was obligatory and unquestionable. Its removal facilitated access to the posterior margins of the lesion and surgical approximation of soft tissues, given that surgical reconstruction techniques were limited at the time. These techniques included soft tissue approximation, skin grafts, adjacent flaps and occasional rehabilitation prostheses.^2^ Pectoralis major myocutaneous flaps were developed in the late 1970s which, together with computed tomography and magnetic resonance imaging systems, led to the analysis of other factors related to mandibular bone involvement, such as the tumor site and its relation to the mandibular bone, the thickness of the mandible, the degree of tumor infiltration into cortical bone, and the use of perioperative studies.^3^, ^4^, ^5^ Difficulties in reaching the deep margins of a tumor due to the mandibular arch were solved by developing mandibulotomies for improved surgical access, enabling reliable resection margins. Many modifications have been proposed, including the midline mandibulotomy for the paramidline osteotomy, which we have adopted based on the premise that the paramedian access allows the distal surgical stump for retraction to be shorter. Furthermore, sectioning the lateral portion of the floor of the mouth while preserving the tongue facilitates recovery of swallowing, and does not reduce the options when approximating soft tissues to reconstruct the mouth.^6^,^7^ We chose angled osteotomies to stabilize bone fragments. Ostheosynthesis was done with titanium miniplates, providing adequate fixation and stabilization without requiring a maxillomandibular fixation, which would impair postoperative local evaluation of the surgical wound. Additionally, titanium miniplates do not interfere with radiotherapy.

The main constraint of the marginal resection technique is the thickness of the mandibular bone. Loss of posterior dentition leads to progressive alveolar bone resorption. The mandibular canal becomes superficial rather than occupying its previous equidistant position from the two mandibular margins. Consequently, tumors may extend to the gingival region, easily reaching the mandibular bone by perineural dissemination. It is also necessary to preserve at least one centimeter of mandibular thickness without which there is a significant risk of stress fracture.^8^

The argument in favor of segmental mandibulectomy for extensive tumors is contradicted by our sample; we included only advanced tumor cases. Furthermore, there was no mandibular bone involvement in segmental resections in the control group. The surgical approach should be based on mandibular bone involvement, readily assessed by imaging methods - particularly computed tomography - available in most treatment centers.^9^ The main treatment failure in both groups was local and regional recurrence, which was 35% in the marginal mandibulectomy group, and 36.4% of the segmental mandibulectomies. One patient died due to the disease and lack of surgical margins in the marginal mandibulectomy group, and another died in the segmental mandibulectomy group.

The correlation between close surgical margins and local recurrence was not significant in our series, since two other patients with inadequate margins are disease-free to this date, and there were deaths in patients with adequate surgical margins.

Regardless of the pathology of surgical margins, other factors are also relevant for prognosis such as inadequate assessments of surgical margins, angiolymphatic and perineural invasion, difficulties to assess residual tumor along the section of the pterygoid muscle that naturally retracts after tumor removal, dissemination to retropharyngeal lymphnodes, and lymphnode metastases, particularly when associated with tumor invasion.^10^ The survival rate of the group treated by marginal mandibulectomy was 55%; the survival rate of the group treated by segmental mandibulectomy was 45%. The p-value was equal to 0.8329 in the comparative analysis. Therefore, the unfavorable prognosis of advanced tumors should not encourage segmental resection, as preservation of part of the mandibular arch did not worsen the survival rate, and preserves function and esthetics. The neck dissection strategy is unrelated to the type of mandibulectomy; in our view there was no technical hindrance against maintaining the monoblock resection associated with the mandibular arch when preparing the access mandibulotomy. Two patients that had been treated by marginal mandibulectomy developed osteoradionecrosis, which is 10% of the group of marginal mandibulectomies and 4.7% of the full series. The use of splints to minimize bone movement may reduce the risk of osteoradionecrosis. Literature does not mention mandibulotomy or mandibulectomy as possible causes of osteoradionecrosis.^6^,^11^ Absence of bone complications in 18 patients belonging to the group treated by marginal mandibulectomy was an encouraging point in favor of the method. Temporomandibular junction dysfunction was found only in patients treated by segmental resection where the absence of the mandibular arch, together with sectioning of the pterygoid and temporal muscles, lead to a change in the rotation axis of the contralateral condyle. This may cause severe pain that irradiates to the temporal and cervical regions, frequently mimicking local recurrence.

Reconstruction using osteomyocutanous grafts and microanastomosis is a promising choice for rehabilitation after segmental resection. This technique, however, is limited by coexisting disease, by metabolic depletion of patients with advanced disease that in turn increase the rate of local and systemic complications with potential loss of the graft, by the lack of expert medical teams to carry out vascular microanastomosis, by the increased operating time, and by cost.^12^

## CONCLUSION

Analysis of both groups revealed that preservation of the ascending ramus of the mandible in advanced lesions with no mandibular involvement does not increase the recurrence rate. The presence of advanced lesions, therefore, should not be used as an argument to resect the mandibular arch.
